# Stable evolutionary signal in a Yeast protein interaction network

**DOI:** 10.1186/1471-2148-6-8

**Published:** 2006-01-30

**Authors:** Stefan Wuchty, Albert-Laszlo Barabási, Michael T Ferdig

**Affiliations:** 1 Northwestern Institute on Complexity, Chambers Hall, Northwestern University, 600 Foster Street, Evanston, IL 60202, USA; 2 Department of Physics, 225 Nieuwland Science Hall, University of Notre Dame, Notre Dame, IN 46556, USA; 3 Department of Biology, 107 Galvin Science Hall, University of Notre Dame, Notre Dame, IN 46556, USA

## Abstract

**Background:**

The recently emerged protein interaction network paradigm can provide novel and important insights into the innerworkings of a cell. Yet, the heavy burden of both false positive and false negative protein-protein interaction data casts doubt on the broader usefulness of these interaction sets. Approaches focusing on one-protein-at-a-time have been powerfully employed to demonstrate the high degree of conservation of proteins participating in numerous interactions; here, we expand his 'node' focused paradigm to investigate the relative persistence of 'link' based evolutionary signals in a protein interaction network of *S. cerevisiae *and point out the value of this relatively untapped source of information.

**Results:**

The trend for highly connected proteins to be preferably conserved in evolution is stable, even in the context of tremendous noise in the underlying protein interactions as well as in the assignment of orthology among five higher eukaryotes. We find that local clustering around interactions correlates with preferred evolutionary conservation of the participating proteins; furthermore the correlation between high local clustering and evolutionary conservation is accompanied by a stable elevated degree of coexpression of the interacting proteins. We use this conserved interaction data, combined with *P. falciparum */Yeast orthologs, as proof-of-principle that high-order network topology can be used comparatively to deduce local network structure in non-model organisms.

**Conclusion:**

High local clustering is a criterion for the reliability of an interaction and coincides with preferred evolutionary conservation and significant coexpression. These strong and stable correlations indicate that evolutionary units go beyond a single protein to include the interactions among them. In particular, the stability of these signals in the face of extreme noise suggests that empirical protein interaction data can be integrated with orthologous clustering around these protein interactions to reliably infer local network structures in non-model organisms.

## Background

An ambitious goal of contemporary proteome research is the elucidation of the structure, interactions and functions of the proteins that constitute cells and organisms. During the last few years, large-scale efforts have unraveled the complex web of protein interactions in simple organisms such as *H. pylori *[1], *E. coli *[2] and *S. cerevisiae *[3-7]. Most recently, attention has focused on the first protein interaction maps of complex multicellular organisms such as *C. elegans *[8] and *D. melanogaster *[9]. Although these organisms vary extensively in their complexity, corroborative evidence points to a series of simple organizing principles that characterize all complex protein interaction networks [10]. The most dramatic of these is their scale-free nature [11,12], highlighting a small number of highly connected proteins which secure the integrity and connectivity among modules [13,14] that are discernible, yet topologically overlapping, clusters of densely interconnected protein groups sharing well-defined functions [10,15-18]. A crucial biological corollary of this ubiquitous network organization is the observation that hubs exhibit an elevated propensity to be simultaneously conserved in evolution and are essential for survival [13,19,20]. This role of highly connected proteins is further indicated by a considerable degree of sequence conservation [21-25]. Similarly, cohesively bound modules have been conserved as a whole, suggesting the presence of evolutionary relevant building blocks [26-28]. This hypothesis is further supported by the observation that proteins belonging to a certain module tend to be coexpressed [29] and coregulated [30]. These particular results are utilized for the comparison of protein pathways of various organisms [31], modeling of interactomes [32,33] and prediction of protein functions [34].

These insights have fundamental implications for our understanding of biological processes and potential applications; however the severe error-proneness of methods for the determination of protein interactions casts doubt on the integrity of such datasets. For example, an estimate of the accuracy of protein interactions in *S. cerevisae *uncovered a startling false negative rate of 90%, and a 50% false positive error rate [35].

Despite incoherences in the determination of protein interactions and orthologs, we observe that extensive information remains in the topology of a protein interaction network. In particular, even tremendous experimental noise does not bury the strong evolutionary signal that highly connected nodes in an interaction web of Yeast proteins are preferably conserved in higher eukaryotes. Accounting for interactions between pairs of Yeast proteins, we find that the reliability of an interaction as indicated by a high degree of local clustering around interactions is accompanied by an elevated propensity for the corresponding proteins to be evolutionary conserved. In addition, we observe that such interactions are preferably coexpressed in both the reference and a target organism, suggesting that conservation occurs not only on the level of individual proteins but also on the level of their interactions. The observation that such link-based evolutionary signals prevail in the topology of an otherwise extremely noisy protein interaction network indicates a novel way to uncover protein interactions in any organism for which orthologs can be identified from sequence data.

## Results

As a basis of our considerations we utilized a protein-protein interaction network of *S. cerevisae *from the DIP database [36], providing 3, 833 proteins embedded in 11, 942 interactions. We labeled pairs of proteins as orthologous to each other as of the InParanoid database [37] that relates proteins of *S. cerevisiae *to complete protein sets of various higher eukaryotes, allowing us to utilize 1, 928 Yeast proteins with putative orthologs in *H. sapiens*, 2,073 in *A. thaliana*, 1, 885 in *C. elegans*, 1, 885 in *M. musculus *and 1,631 in *D. melanogaster.*

### Evolutionary retention of single proteins

**Figure 1:**
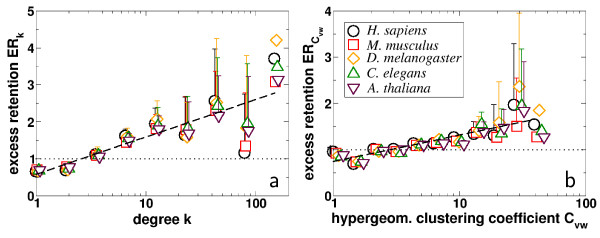
**(a) **Pooling proteins according to their level of interaction *k *we determined the excess retention *ER *of these grouped protein (pair)s that have orthologs in *H. sapiens, M. musculus, D. melanogaster, C. elegans *and *A. thaliana*. Averaging these *k *depending values of *ER*_*k *_in bins of logarithmic size we observe clear logarithmic trends. **(b) **Analogously, we pooled interacting protein pairs *v, w *of Yeast according to their hypergeometric clustering coefficient *C*_*vw *_and determined the excess retention *ER *in these groups of protein pairs that both have orthologs in the aforementioned higher eukaryotes. Pooling these *C*_*vw *_dependent values of *ER *in bins of logarithmically increasing size we observe clear logarithmic trends again. In both cases, significant Pearson's and Spearman's rank correlation coefficients [see Additional file 1] support our conclusion that not only highly interacting proteins (as exemplified by a large *k*) are predominately preserved but also interacting protein pairs which are embedded in a highly cohesive neighborhood (as exemplified by a high *C*_*vw*_). Error bars indicate the standard deviations from the mean excess retention in each bin.

Utilizing these data sets we recently uncovered a correlation between a Yeast proteins level of interaction and its propensity to be evolutionary conserved [20]. Pooling all proteins into groups according to their connectivity *k *we determine the respective fraction of orthologs in each group. As a null-hypothesis we assume a random distribution of orthologs that is quantified by the fraction of proteins with an ortholog in a target eukaryote and the total number of proteins present in the underlying Yeast protein interaction network. The degree dependent orthologous excess retention, *ER*_*k*_, defined as the ratio of ortholog fractions in *k *dependent groups of proteins and fractions of randomly distributed orthologous proteins reflects the dependence of evolutionary protein conservations as function of the proteins connectivity. Logarithmically binning the *k*-dependent values of *ER*_*k *_the averages in each bin show a clear and systematic trend toward preferred conservation of proteins that interact on a high level (Fig. 1a). Significant Pearson's and Spearman's rank coefficients support our qualitative observations [see Additional file 1].

### Evolutionary retention of interacting pairs of proteins

While we find that the conservation of single proteins is a function of connectedness we wonder if topology also contains such evolutionary signals on the level of interactions. Because proteins which are placed in cohesive areas (i.e. modules) tend to be evolutionary conserved we wonder if their interactions are conserved too. We utilize a link-based clustering coefficient that reflects the degree of clustering of an interaction's immediate network neighborhood, a topological measure that allows for correlations between local clustering and the actual reliability of observed interactions [38]. Similar to the single protein case, we grouped all interactions according to their hypergeometric clustering coefficient *C*_*vw *_and determined the respective fraction of interacting pairs that are fully conserved as putative orthologs in each bin. In the absence of a correlation between evolutionary conservation and an interactions placement in the network the ratio of the *C*_*vw*_-dependent and random fractions of orthologous protein pairs – defined as the interaction based excess retention ERCvw
 MathType@MTEF@5@5@+=feaafiart1ev1aaatCvAUfKttLearuWrP9MDH5MBPbIqV92AaeXatLxBI9gBaebbnrfifHhDYfgasaacH8akY=wiFfYdH8Gipec8Eeeu0xXdbba9frFj0=OqFfea0dXdd9vqai=hGuQ8kuc9pgc9s8qqaq=dirpe0xb9q8qiLsFr0=vr0=vr0dc8meaabaqaciaacaGaaeqabaqabeGadaaakeaacqWGfbqrcqWGsbGudaWgaaWcbaGaem4qam0aaSbaaWqaaiabdAha2jabdEha3bqabaaaleqaaaaa@334B@ (see Materials and Methods) – would be unity. Logarithmically binning all interactions according to their local degree of clustering *C*_*vw *_and determining the average excess retention ERCvw
 MathType@MTEF@5@5@+=feaafiart1ev1aaatCvAUfKttLearuWrP9MDH5MBPbIqV92AaeXatLxBI9gBaebbnrfifHhDYfgasaacH8akY=wiFfYdH8Gipec8Eeeu0xXdbba9frFj0=OqFfea0dXdd9vqai=hGuQ8kuc9pgc9s8qqaq=dirpe0xb9q8qiLsFr0=vr0=vr0dc8meaabaqaciaacaGaaeqabaqabeGadaaakeaacqWGfbqrcqWGsbGudaWgaaWcbaGaem4qam0aaSbaaWqaaiabdAha2jabdEha3bqabaaaleqaaaaa@334B@ in each bin we identify a significant and systematic trend of proteins engaged in highly clustered interactions to be preferably evolutionary conserved [Fig. 1b, see Additional file 1]. These link-based observations are not only consistent with previous node-based results but also allow to suggest that standard single-node measurements of evolutionary conservation can be extended to their neighboring links. This evolutionary corollary indicates that not only single proteins are a target of evolution but also the interactions between conserved proteins.

### Perturbation analysis

To demonstrate this gain of evolutionary information, we simulated the impact of extremely high false negatives rates of protein interactions by removing up to 70% of experimentally determined links between randomly selected protein pairs. Additionally, to address the effects of false positives, we randomly distributed up to 70% more interactions than were previously identified in the original Yeast network.

**Figure 2:**
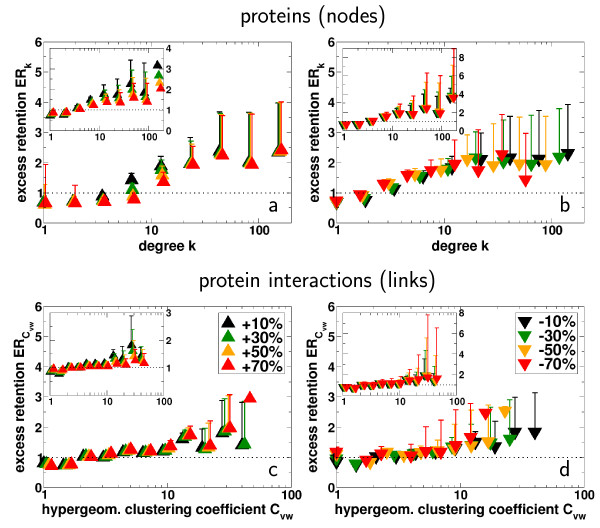
**(a) **In order to assess the impact of severely inconsistent protein interaction data, we removed 10–70% of interactions between randomly selected protein pairs, mimicking false negatives. **(b) **Simulating the effects of false positives, we randomly added 10–70% more interactions than originally present in the network. In each case, we averaged the degree dependent excess retention of interacting proteins *ER*_*k *_that have orthologs in *C. elegans *over 1, 000 different samples. Analogously, we assessed the consequences of false negative orthologs by eliminating 10–70% of the proteins present in the set of worm orthologs ((a) inset). Mimicking the presence of false positive orthologs we labeled 10–70% more proteins as orthologs in worm that were originally present ((b) inset). Analogously, we test the robustness of the trend that highly clustered interactions (as exemplified by the hypergeometric clustering coefficient *C*_*vw*_) are indeed predominantly conserved (as exemplified by a link based excess retention ERCvw
 MathType@MTEF@5@5@+=feaafiart1ev1aaatCvAUfKttLearuWrP9MDH5MBPbIqV92AaeXatLxBI9gBaebbnrfifHhDYfgasaacH8akY=wiFfYdH8Gipec8Eeeu0xXdbba9frFj0=OqFfea0dXdd9vqai=hGuQ8kuc9pgc9s8qqaq=dirpe0xb9q8qiLsFr0=vr0=vr0dc8meaabaqaciaacaGaaeqabaqabeGadaaakeaacqWGfbqrcqWGsbGudaWgaaWcbaGaem4qam0aaSbaaWqaaiabdAha2jabdEha3bqabaaaleqaaaaa@334B@) toward the presence of false negative **(c) **and false positive **(d) **interactions and orthologs (insets). In each case, we observe that the initial (empirically derived) ascending trend prevails, results which are further supported by strong and significant Pearson's and Spearman's rank correlation coefficients and Kolmogorov-Smirnov scores [see Additional file 1]. Error bars indicate the standard deviations from the mean excess retention in each bin.

Because there are no significant differences in the distributions of organism-specific excess retention in Fig. 1, we examine orthologs of *C. elegans *as a representative comparative set for these analyzes. After generating 1, 000 different realizations to each case of incomplete false data, we determine the excess retention *ER *of proteins and their interactions that have orthologs in *C. elegans *according to their degree *k *and hypergeometric clustering coefficient *C*_*vw*_. Determining the average excess retention in bins of increasing logarithmic size we find that the relationship between excess retention, level of interaction and local clustering is widely unaltered (single proteins: Figs. 2a,b; protein interactions: Figs. 2c,d). Addressing the statistical significance of these results we determined correlation coefficients (Pearson's *r *and Spearman's rank *ρ*) and performed Kolmogorov-Smirnov tests, recovering significant similarities between the original and perturbed distributions [see Additional file 1].

Moreover, to represent missed orthologs, we randomly eliminated up to 70% from the set of Yeast proteins that have an ortholog in *C. elegans*. In turn, we randomly labeled up to 70% more proteins as orthologs in *C. elegans *than were previously present in the initial set. Sampling 1, 000 different realizations each, we calculated the excess retention according to the proteins degree *k *and local clustering around each interaction *C*_*vw*_. Logarithmically binning the results thus obtained we averaged the excess retention of orthologous proteins in each bin, allowing us to find that the introduction of noise on the level of orthologs determination does not alter our initial observations (single proteins: insets Figs. 2a,b; protein interactions: insets Figs. 2c,d). Significantly similar correlation coefficients and Kolmogorov-Smirnov scores [see Additional file 1] support our conclusions.

### Clustering, coexpression and evolutionary conservation

**Figure 3:**
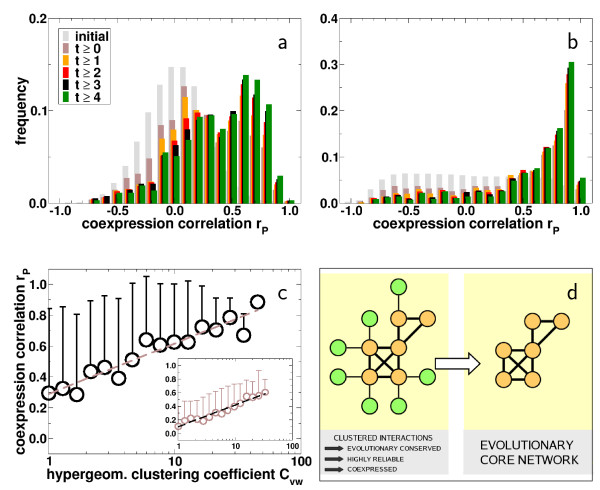
**(a) **Accounting for the expression coefficients of all protein pairs in Yeast, we observe a bell shaped frequency distribution of the expression correlation coefficient *r*_*P*_, peaking around 0.0 (initial). Focusing on interacting pairs of Yeast proteins that both have an ortholog in *P. falciparum *and score above a certain threshold *t *of the respective hypergeometric clustering coefficient *C*_*vw *_we observe shifted frequency distributions of the expression correlation coefficients *r*_*P*_. This observation is further indicated by significant Students t-test scores [see Additional file 1]. **(b) **Assuming that those interactions constituted by Yeast proteins with an ortholog are conserved as well in *P. falciparum*, we determined frequency distributions of expression correlation coefficients *r*_*P *_in this organism. Similarly to (a), we find significant shifts toward enforced coexpression if we focus on conserved interactions in Plasmodium that were embedded in an increasingly cohesive neighborhood in Yeast. Comparing our results to a background distribution of coexpression correlations of all protein pairs in *P. falciparum *Students t-test scores indicate the significance of our results [see Additional file 1]. **(c) **The significant shifts toward elevated levels of coexpression allow us to assume that there exists a pronounced correlation between the local cohesiveness of an interaction and the tendency that the involved proteins are coexpressed. Logarithmically binning data points according to their hypergeometric clustering coefficient *C*_*vw *_in Yeast, we determined the mean expression correlation *r*_*P *_in each bin, allowing us to observe a positive and significant trend that interacting Yeast proteins having an ortholog in *P. falciparum *are increasingly coexpressed if they are placed in a cohesive neighborhood (inset, Pearson's *r *= 0.38, *P *= 8.8 × 10^-44^, Spearman's rank *ρ *= 0.41, *P *= 2.8 × 10^-47^). Similarly, we observe qualitatively the same trend considering interactions in *P. falciparum *that have been inferred from Yeast protein interactions which have an ortholog in *P. falciparum *(Pearson's *r *= 0.25, *P *= 1.9 × 10^-27^, Spearman's rank *ρ *= 0.27, *P *= 2.1 × 10^-30^). Error bars indicate the standard deviation from the mean coexpression coefficient in each bin. **(d) **Concluding, we observe a perturbation persistent coincidence of (i) coexpression of interacting proteins, (ii) an enhanced clustering of their immediate neighborhood and (iii) their elevated tendency to be evolutionary conserved (yellow circles) in *S. cerevisiae*. Since high clustering around a certain protein interaction coincide well with an elevated reliability the integration of knowledge about the local clustering of an interacting pair of conserved proteins and their tendency to be coexpressed can be used to infer evolutionary core protein interactions in other organisms for which orthologs can be identified.

The observation that highly clustered links between evolutionarily conserved proteins are reliable and stable toward severe perturbation enhances our expectation that an elevated degree of coexpression of interacting proteins will retain this relationship as well. In particular, a strong coexpression signal of the orthologs of proteins that embrace the interactions in question would strongly indicate the actual presence of the interaction in a reference and target organism. As a test case, we extend our investigations to the malaria parasite *Plasmodium falciparum*, a single celled organism that has 895 putative orthologous proteins with Yeast. Elucidating those Yeast interactions between yeast proteins conserved in *P. falciparum*, we find a web of 3, 071 interactions among 659 proteins in *P. falciparum*. To evaluate the quality of these inferred interactions we utilized a comprehensive set of *P. falciparum *specific coexpression data [39] to calculate Pearson's correlation coefficients *r*_*P *_for the inferred protein interactions. In the same way, we utilized an extensive set of Yeast coexpression data [40] to investigate the coexpression tendency of those interacting Yeast proteins that served as the template for the inferred interactions in Plasmodium. In both cases, we use the Yeast specific *C*_*vw *_values as an approximate measure of an interaction's reliability. Focusing on interactions that score above increasing thresholds of *C*_*vw*_, we observe a strong shift toward coexpression of the considered protein pairs (Fig. 3a,b). The difference between the individual coexpression patterns is further indicated by significant Students t-test scores when the *C*_*vw *_dependent distributions are compared to a background distribution of coexpression coefficients of all protein pairs in the considered organism [see Additional file 1]. The significant shifts toward elevated levels of coexpression identify a pronounced correlation between the local cohesiveness of an interaction and the tendency that the involved proteins are coexpressed. The determination of mean coexpression coefficients *r*_*P *_of interactions that have been logarithmically grouped according to their *C*_*vw *_allows us to find a statistically significant trend toward elevated levels of coexpression of conserved yeast interactions that are placed in highly clustered neighborhood (inset, Fig. 3c). Although we adopt measurements of the local cohesiveness around links *C*_*vw *_from Yeast, we find that the corresponding interactions in Plasmodium exhibit a similar trend (Fig. 3c). Remarkably, the latter distribution exhibits better and statistically more significant correlation coefficients than its template in Yeast [see Additional file 1].

In the same way we investigated the stability of the interactions propensity to be evolutionary conserved, we checked for the robustness of the obtained correlation between local clustering and coexpression. Mimicking the presence of false positive/negative links we randomly eliminated/added up to 70 % of interactions in the Yeast interaction network. Recalculating the hypergeometric clustering coefficient for each of 1,000 runs, we grouped all interacting pairs of Yeast proteins with an ortholog in *P. falciparum *according to *C*_*vw *_in bins of logarithmically increasing size. Averaging over the respective coexpression correlation coefficient *r*_*P *_of all Yeast interactions in each bin, we observe that the initial ascending trend prevails [see Fig. 1ab of Additional file 1]. Assuming that all interactions between proteins that have an ortholog in Plasmodium were conserved we repeated this procedure by superimposing and averaging over the respective coexpression correlation values of Plasmodium. Similar to the Yeast specific case, we observe the same qualitative trends [see Fig. 2ab of Additional file 1]. Significant correlation coefficients and Kolmogorov-Smirnov scores support our observations of our findings. In the same way, we simulated the presence of false positive /negative orthologs by eliminating/adding up to 70% of orthologs in *P. falciparum*. Averaging over 1,000 runs each, we determined the average coexpression coefficient in each bin utilizing Plasmodium and Yeast specific coexpression data. In each case, we find that the original trend of high local clustering around interactions coincides with an increased propensity to be coexpressed strongly prevails [see Figs. 1, 2bc of Additional file 1], observations that are supported by significant correlation coefficients and Kolmogorov-Smirnov scores [see Additional file 1].

## Discussion & conclusion

Extending a previous study indicating that highly interacting proteins are predominantly conserved in evolution we generalize the concept that evolutionary signals are carried by the topology of the underlying protein interaction network. In particular, a protein's propensity to be conserved while interacting with a high number of partners – a node-based evolutionary signal – has a link based counterpart, as indicated by the propensity of interacting proteins to be evolutionary conserved with increasing local clustering around the interaction in question. Although the obtained correlations are significant, the alarmingly high error rates in the determination of protein interactions cast doubt on the obtained results.

By focusing on perturbation events on node and interaction levels, we observe that extreme error rates of both protein interactions and orthologs do not ablate the evolutionary signal carried by the network structure. The introduction of noise at the node, by simulation of inconsistent determination of orthologs, does not override the preference of highly connected nodes to be evolutionary conserved; as theoretically predicted, random perturbations will rarely affect a hub in a scale-free network [41]. The low probability that a hub is hit by a random perturbation event also explains that interacting proteins that are placed in a highly clustered environment retain their evolutionary signal. Indeed, the definition of the hypergeometric clustering coefficient assures a high score for interacting proteins that share a lot of their interaction partners.

On an interaction level, we observe that the massive insertion/deletion of links does not obliterate the local structure of networks as indicated by the stable preference of highly connected proteins and protein pairs that are embedded in a well clustered neighborhood to be evolutionary conserved. In particular, we conclude that insertion/deletion of random links on average impact sparsely connected parts of the networks much more than densely connected ones; indeed, loss of information in highly clustered neighborhoods and highly connected hubs would require massive, targeted deletion/insertion of links to obliterate their local structure. Therefore, the observation that links which are placed in a highly clustered neighborhood are highly reliable [38] is nested in our observation that highly clustered neighborhoods compensate severe random perturbations much better than sparsely connected ones.

While our results allow us to conclude that degree alone is a robust indicator for a proteins propensity to be evolutionary conserved, the inherent topological robustness of locally clustered links emphasizes the emergent role of cohesive areas [26] as mediators of evolutionary information. In the simplest case, we confirmed that not only single proteins are a potential target of evolution but interaction among them can be potentially conserved as well. As a strong indicator that an interaction indeed has been conserved, the correlation between high local clustering and evolutionary conservation is accompanied by a stable elevated degree of coexpression of the interacting proteins in both a model and target organism. Superimposing the extreme error rates simulating the incoherent determination of orthologs and interactions as well we see that trends in both the model and target organism prevail, strongly indicating that evolution also happens on the level of interactions and putative bundles of interactions.

Although we utilized very noisy and inconsistent data of protein interactions and putative orthologs, we see that high connectivity and high clustering on average harbor significantly more evolutionary relevant information that sparsely connected and clustered areas. The coincidence of (i) high local clustering around highly reliable interactions of proteins, (ii) their propensity to be evolutionary conserved, (iii) their tendency to be coexpressed even in the face of tremendous experimental noise sketches a hypothetical framework to infer an evolutionary core of single protein-protein interactions by elucidating interacting proteins of a reference organism that have orthologs in the targeted organism. The quality of an interaction is assessed by calculating the corresponding hypergeometric clustering coefficient. Choosing the highest scoring – thus most reliable – ortholog interaction allows the selection of a core interaction network in the targeted organism. Unlike our case, where evolutionary relationships between proteins were approximated by similarity searches, the quality of predicted interactions will be enhanced by utilizing more sophisticated methods (such as tree-base methods) which allow a more reliable assignment of orthology. Finally, the cross-validation with high resolution coexpression data can refine specific protein-protein interaction subnetworks, allowing for checks of the actual presence of a proposed interaction. Ultimately, such a framework would allow a first insight into evolutionary conserved parts in interactomes of organism for which no interaction data currently exists.

## Methods

### Protein interactions

As a source of protein interactions we chose the DIP database [36] which provides a set of manually curated protein-protein interactions in the organism *S. cerevisiae*. The current version contains 3, 833 proteins involved in 11, 942 interactions derived from combined, non-overlapping data which are mostly obtained from the high-throughput application of the two-hybrid method.

### Assignment of orthology

Orthologs are genes in different species that originate from a single gene in the last common ancestor of these species. Such genes often have retained identical biological roles in present day organisms, indicated by a high degree of sequence homology. Unfortunately, orthology analysis between organisms is often difficult and error prone because of large numbers of paralogs within protein families. As a source of reliable and robust information about orthologous relationships between proteins in different species we utilized the InParanoid database [37,42] which provides putative orthologous sequence information for *S. cerevisiae *and numerous other organisms. The algorithm for assigning orthologous relationships is based on pairwise similarity scores which are by default calculated with the BLASTP program. Best pairwise hits between the proteomes of two species are seeds – labeled as the main ortholog groups – of orthologous protein sequence clusters. In a further step, other sequences are added to this group if they are closely homologous to one of the main orthologs, members of orthologous groups which are called in-paralogs. In a final quality checking step, confidence values for each ortholog and in-paralog is determined allowing the detection of putative orthologous relationships that has been only reliably possible by multiple alignments and phylogenetic trees previously [37]. In our study, we considered the main ortholog pairs of each orthologous group as sequences that are putatively orthologous to each other allowing us to obtain 1, 928 Yeast proteins with orthologs in *H. sapiens*, 2,073 in *A. thaliana*, 1, 885 in *C. elegans*, 1, 885 in *M. musculus*, 1,631 in *D. melanogaster *and 895 in *P. falciparum.*

### Hypergeometric clustering coefficient

Recently, a network topology based approach uncovered a remarkable correlation between enhanced quality of protein interactions and the degree of clustering of their immediate network neighborhood [38]. Considering a protein-protein interaction network with *N *nodes, we define the hypergeometric clustering coefficient as

Cvw=−log⁡∑i=|N(v)∩N(w)|min⁡(|N(v)|,|N(w)|)(|N(v)|i)(N−|N(v)||N(w)|−i)(|N(w)|N)     (1)
 MathType@MTEF@5@5@+=feaafiart1ev1aaatCvAUfKttLearuWrP9MDH5MBPbIqV92AaeXatLxBI9gBaebbnrfifHhDYfgasaacH8akY=wiFfYdH8Gipec8Eeeu0xXdbba9frFj0=OqFfea0dXdd9vqai=hGuQ8kuc9pgc9s8qqaq=dirpe0xb9q8qiLsFr0=vr0=vr0dc8meaabaqaciaacaGaaeqabaqabeGadaaakeaafaqaaeqacaaabaGaem4qam0aaSbaaSqaaiabdAha2jabdEha3bqabaGccqGH9aqpcqGHsislcyGGSbaBcqGGVbWBcqGGNbWzaeaadaaeWbqaamaalaaabaWaaeWaaeaadaWfqaqaamaaemaabaGaemOta40aaeWaaeaacqWG2bGDaiaawIcacaGLPaaaaiaawEa7caGLiWoaaSqaaiabdMgaPbqabaaakiaawIcacaGLPaaadaqadaqaamaaxababaGaemOta4KaeyOeI0YaaqWaaeaacqWGobGtdaqadaqaaiabdAha2bGaayjkaiaawMcaaaGaay5bSlaawIa7aaWcbaWaaqWaaeaacqWGobGtdaqadaqaaiabdEha3bGaayjkaiaawMcaaaGaay5bSlaawIa7aiabgkHiTiabdMgaPbqabaaakiaawIcacaGLPaaaaeaadaqadaqaamaaxacabaWaaqWaaeaacqWGobGtdaqadaqaaiabdEha3bGaayjkaiaawMcaaaGaay5bSlaawIa7aaWcbeqaaiabd6eaobaaaOGaayjkaiaawMcaaaaaaSqaaiabdMgaPjabg2da9maaemaabaGaemOta40aaeWaaeaacqWG2bGDaiaawIcacaGLPaaacqGHPiYXcqWGobGtdaqadaqaaiabdEha3bGaayjkaiaawMcaaaGaay5bSlaawIa7aaqaaiGbc2gaTjabcMgaPjabc6gaUnaabmaabaWaaqWaaeaacqWGobGtdaqadaqaaiabdAha2bGaayjkaiaawMcaaaGaay5bSlaawIa7aiabcYcaSmaaemaabaGaemOta40aaeWaaeaacqWG3bWDaiaawIcacaGLPaaaaiaawEa7caGLiWoaaiaawIcacaGLPaaaa0GaeyyeIuoakiaaxMaacaWLjaWaaeWaaeaacqaIXaqmaiaawIcacaGLPaaaaaaaaa@8AF9@

where *N*(*x*) represents the neighborhood of a vertex *x*. Given fixed neighborhood sizes *N*(*v*) and *N*(*w*) of proteins *v *and *w*, the hypergeometric clustering coefficient increases with elevated overlap between the protein's neighborhoods. Provided that the neighborhoods are independent, the summation can be interpreted as a *p *value, reflecting the probability of obtaining a number of mutual neighbors between proteins *v *and *w *at or above the observed number by chance.

### Orthologous excess retention

According to their hypergeometric clustering coefficient *C*_*vw *_of the interactions they are involved in, we grouped all interactions in groups of same *C*_*vw *_that have been rounded to integers. For each group of NCvw
 MathType@MTEF@5@5@+=feaafiart1ev1aaatCvAUfKttLearuWrP9MDH5MBPbIqV92AaeXatLxBI9gBaebbnrfifHhDYfgasaacH8akY=wiFfYdH8Gipec8Eeeu0xXdbba9frFj0=OqFfea0dXdd9vqai=hGuQ8kuc9pgc9s8qqaq=dirpe0xb9q8qiLsFr0=vr0=vr0dc8meaabaqaciaacaGaaeqabaqabeGadaaakeaacqWGobGtdaWgaaWcbaGaem4qam0aaSbaaWqaaiabdAha2jabdEha3bqabaaaleqaaaaa@3230@ proteins, the fraction of interacting pairs of proteins that both have an ortholog in an other organism is defined as eCvw,o=nCvw,o/NCvw
 MathType@MTEF@5@5@+=feaafiart1ev1aaatCvAUfKttLearuWrP9MDH5MBPbIqV92AaeXatLxBI9gBaebbnrfifHhDYfgasaacH8akY=wiFfYdH8Gipec8Eeeu0xXdbba9frFj0=OqFfea0dXdd9vqai=hGuQ8kuc9pgc9s8qqaq=dirpe0xb9q8qiLsFr0=vr0=vr0dc8meaabaqaciaacaGaaeqabaqabeGadaaakeaacqWGLbqzdaWgaaWcbaGaem4qam0aaSbaaWqaaiabdAha2jabdEha3bqabaWccqGGSaalcqWGVbWBaeqaaOGaeyypa0JaemOBa42aaSbaaSqaaiabdoeadnaaBaaameaacqWG2bGDcqWG3bWDaeqaaSGaeiilaWIaem4Ba8gabeaakiabc+caViabd6eaonaaBaaaleaacqWGdbWqdaWgaaadbaGaemODayNaem4DaChabeaaaSqabaaaaa@4434@. In the absence of a correlation between evolutionary conservation of interacting protein pairs and their position in the network, eCvw,o
 MathType@MTEF@5@5@+=feaafiart1ev1aaatCvAUfKttLearuWrP9MDH5MBPbIqV92AaeXatLxBI9gBaebbnrfifHhDYfgasaacH8akY=wiFfYdH8Gipec8Eeeu0xXdbba9frFj0=OqFfea0dXdd9vqai=hGuQ8kuc9pgc9s8qqaq=dirpe0xb9q8qiLsFr0=vr0=vr0dc8meaabaqaciaacaGaaeqabaqabeGadaaakeaacqWGLbqzdaWgaaWcbaGaem4qam0aaSbaaWqaaiabdAha2jabdEha3bqabaWccqGGSaalcqWGVbWBaeqaaaaa@34A5@ has the general *C*_*vw*_-independent value *e*_*o *_= *n*_*o*_/*N*, where *n*_*o *_is the total number of interactions between Yeast proteins that have an ortholog, and *N *is the total number of Yeast protein interactions in the underlying network. Thus, we define the clustering-dependent excess retention of such proteins as ERCvw,o=eCvw,o/eo
 MathType@MTEF@5@5@+=feaafiart1ev1aaatCvAUfKttLearuWrP9MDH5MBPbIqV92AaeXatLxBI9gBaebbnrfifHhDYfgasaacH8akY=wiFfYdH8Gipec8Eeeu0xXdbba9frFj0=OqFfea0dXdd9vqai=hGuQ8kuc9pgc9s8qqaq=dirpe0xb9q8qiLsFr0=vr0=vr0dc8meaabaqaciaacaGaaeqabaqabeGadaaakeaacqWGfbqrcqWGsbGudaWgaaWcbaGaem4qam0aaSbaaWqaaiabdAha2jabdEha3bqabaWccqGGSaalcqWGVbWBaeqaaOGaeyypa0Jaemyzau2aaSbaaSqaaiabdoeadnaaBaaameaacqWG2bGDcqWG3bWDaeqaaSGaeiilaWIaem4Ba8gabeaakiabc+caViabdwgaLnaaBaaaleaacqWGVbWBaeqaaaaa@4271@ which has the *C*_*vw*_-independent value ERCvw,o=1
 MathType@MTEF@5@5@+=feaafiart1ev1aaatCvAUfKttLearuWrP9MDH5MBPbIqV92AaeXatLxBI9gBaebbnrfifHhDYfgasaacH8akY=wiFfYdH8Gipec8Eeeu0xXdbba9frFj0=OqFfea0dXdd9vqai=hGuQ8kuc9pgc9s8qqaq=dirpe0xb9q8qiLsFr0=vr0=vr0dc8meaabaqaciaacaGaaeqabaqabeGadaaakeaacqWGfbqrcqWGsbGudaWgaaWcbaGaem4qam0aaSbaaWqaaiabdAha2jabdEha3bqabaWccqGGSaalcqWGVbWBaeqaaOGaeyypa0JaeGymaedaaa@3792@ for a random distribution of orthologous proteins [20]. Basically, we applied the same framework for single proteins, by grouping them according to their degree *k*. For each group of *N*_*k *_proteins, the fraction of proteins that also have an ortholog is defined as *e*_*k*,*o *_= *n*_*k*,*o*_/*N*_*k*_. Analogously, the node based excess retention *ER*_*k *_is defined as *ER*_*k *_= *e*_*k*,*o*_/*E*_*k*_, where *E*_*k *_is the ratio of all proteins with an ortholog in the whole network.

### Coexpression data

To evaluate the quality of these inferred interactions we utilized a comprehensive set of Plasmodium specific [39] and Yeast specific [40] coexpression data. In each dataset, we utilized the expression profiles to determine the respective Pearson's correlation coefficient *r*_*P *_for each interacting pair of proteins.

### Logarithmic binning

To guarantee balanced sampling of our distributions we generally use logarithmic binning of the respective *x*-axis, a procedure for curve estimation that corrects for the skewed nature of the scale-free distribution.

On a logarithmic scale, we define the bin size Δ=1Nlog⁡(ba)
 MathType@MTEF@5@5@+=feaafiart1ev1aaatCvAUfKttLearuWrP9MDH5MBPbIqV92AaeXatLxBI9gBaebbnrfifHhDYfgasaacH8akY=wiFfYdH8Gipec8Eeeu0xXdbba9frFj0=OqFfea0dXdd9vqai=hGuQ8kuc9pgc9s8qqaq=dirpe0xb9q8qiLsFr0=vr0=vr0dc8meaabaqaciaacaGaaeqabaqabeGadaaakeaacqqHuoarcqGH9aqpdaWcaaqaaiabigdaXaqaaiabd6eaobaacyGGSbaBcqGGVbWBcqGGNbWzdaqadaqaamaalaaabaGaemOyaigabaGaemyyaegaaaGaayjkaiaawMcaaaaa@398C@, where *N *corresponds to the selected number of bins. Values *a *and *b *refer to the minimal and maximal value of data points on the *x*-axis, *b *= max_*i*_(*x*_*i*_) and *a *= min_*i*_(*x*_*i*_). Thus, *n*_*i *_= log⁡(xia)
 MathType@MTEF@5@5@+=feaafiart1ev1aaatCvAUfKttLearuWrP9MDH5MBPbIqV92AaeXatLxBI9gBaebbnrfifHhDYfgasaacH8akY=wiFfYdH8Gipec8Eeeu0xXdbba9frFj0=OqFfea0dXdd9vqai=hGuQ8kuc9pgc9s8qqaq=dirpe0xb9q8qiLsFr0=vr0=vr0dc8meaabaqaciaacaGaaeqabaqabeGadaaakeaacyGGSbaBcqGGVbWBcqGGNbWzdaqadaqaamaalaaabaGaemiEaG3aaSbaaSqaaiabdMgaPbqabaaakeaacqWGHbqyaaaacaGLOaGaayzkaaaaaa@36B8@/*Δ*, *n*_*i *_∈ [0, *N *- 1] reflects the number of the bin we assign a data point with a *x*_*i *_coordinate. Representing the *n*_*i*_th bin on the *x*-axis, we place xni
 MathType@MTEF@5@5@+=feaafiart1ev1aaatCvAUfKttLearuWrP9MDH5MBPbIqV92AaeXatLxBI9gBaebbnrfifHhDYfgasaacH8akY=wiFfYdH8Gipec8Eeeu0xXdbba9frFj0=OqFfea0dXdd9vqai=hGuQ8kuc9pgc9s8qqaq=dirpe0xb9q8qiLsFr0=vr0=vr0dc8meaabaqaciaacaGaaeqabaqabeGadaaakeaacqWG4baEdaWgaaWcbaGaemOBa42aaSbaaWqaaiabdMgaPbqabaaaleqaaaaa@3149@ at the end of each bin using xni=aeΔ(ni+1)
 MathType@MTEF@5@5@+=feaafiart1ev1aaatCvAUfKttLearuWrP9MDH5MBPbIqV92AaeXatLxBI9gBaebbnrfifHhDYfgasaacH8akY=wiFfYdH8Gipec8Eeeu0xXdbba9frFj0=OqFfea0dXdd9vqai=hGuQ8kuc9pgc9s8qqaq=dirpe0xb9q8qiLsFr0=vr0=vr0dc8meaabaqaciaacaGaaeqabaqabeGadaaakeaacqWG4baEdaWgaaWcbaGaemOBa42aaSbaaWqaaiabdMgaPbqabaaaleqaaOGaeyypa0JaemyyaeMaemyzau2aaWbaaSqabeaacqqHuoardaqadaqaaiabd6gaUnaaBaaameaacqWGPbqAaeqaaSGaey4kaSIaeGymaedacaGLOaGaayzkaaaaaaaa@3CDD@.

The advantage of logarithmic binning is an elevated degree of noise reduction which is dependent on the bin size [41,43]. Although this procedure causes a loss of accuracy, we still uncover the buried trends to a satisfying extent applying our statistical methods on the binned data.

## Supplementary Material

Additional File 1 contains detailed statistical measurements of presented correlations. Furthermore, we show results of the perturbation analysis of coexpression coefficients as briefly addressed in the main paper. Click here for file
