# Supplementary material for: Stable evolutionary signal in a Yeast protein interaction network
Source: BMC Evol Biol. 2006 Jan 30;6:8. doi: 10.1186/1471-2148-6-8 (PMC1395346; doi:10.1186/1471-2148-6-8)
Supplement: Additional File 1 — contains detailed statistical measurements of presented correlations. Furthermore, we show results of the perturbation analysis of coexpression coefficients as briefly addressed in the main paper. [file 1471-2148-6-8-S1.pdf]

Stability of evolutionary signals in a protein interaction network of Yeast - Supplementary Material

Stefan Wuchty[1], Albert-László Barabási[2] and Michael T. Ferdig[3]

[1]Northwestern Institute of Complexity, Northwestern University, Evanston, IL, U.S.A.

[2]Department of Physics, University of Notre Dame, Notre Dame, IN 46556, U.S.A.

[3]Department of Biology, University of Notre Dame, Notre Dame, IN 46556, U.S.A.

organism	$ER_k \sim$	r (P-value)	ρ (P-value)
H. sapiens	$0.43 \ln k$	$0.76\ (4.5 \times 10^{-4})$	$0.81\ (2.1 \times 10^{-2})$
M. musculus	$0.37 \ln k$	$0.77\ (3.6 \times 10^{-3})$	$0.81\ (2.1 \times 10^{-2})$
D. melanogaster	$0.54 \ln k$	$0.88\ (1.0 \times 10^{-4})$	$0.88\ (1.2 \times 10^{-2})$
C. elegans	$0.47 \ln k$	$0.87\ (1.7 \times 10^{-4})$	$0.97\ (6.3 \times 10^{-3})$
A. thaliana	$0.40 \ln k$	$0.86\ (2.2 \times 10^{-4})$	$0.92\ (9.5 \times 10^{-3})$

Table 1. Best logarithmic fit ($ER_{C_{vw}} \sim \alpha \ln C_{vw}$), Pearson's ($r$) and Spearman's rank correlation coefficients (ρ) of distributions that show the dependence of single proteins tendency to have orthologs in *H. sapiens*, *M. musculus*, *D. melanogaster*, *C. elegans* and *A. thaliana* (as exemplified by the excess retention ER) from their level of interaction (as exemplified by the degree k). Values refer to Fig. 1a in the main paper.

organism	$ER_{C_{vw}} \sim$	r (P-value)	ρ (P-value)
H. sapiens	$0.24 \ln C_{vw}$	$0.82\ (2.5 \times 10^{-4})$	$0.97\ (2.1 \times 10^{-3})$
M. musculus	$0.16 \ln C_{vw}$	$0.74\ (2.7 \times 10^{-3})$	$0.94\ (3.1 \times 10^{-3})$
D. melanogaster	$0.35 \ln C_{vw}$	$0.88\ (1.1 \times 10^{-5})$	$0.97\ (2.1 \times 10^{-3})$
C. elegans	$0.25 \ln C_{vw}$	$0.75\ (1.9 \times 10^{-3})$	$0.97\ (3.4 \times 10^{-3})$
A. thaliana	$0.22 \ln C_{vw}$	$0.72\ (4.2 \times 10^{-3})$	$0.93\ (3.4 \times 10^{-3})$

Table 2. Best logarithmic fit ($ER_{C_{vw}} \sim \alpha \ln C_{vw}$), Pearson's ($r$) and Spearman's rank correlation coefficients (ρ) of distributions that show the dependence of the interacting proteins tendency to have orthologs in *H. sapiens*, *M. musculus*, *D. melanogaster*, *C. elegans* and *A. thaliana* (as exemplified by the excess retention ER) from the cohesiveness around interactions (as exemplified by the hypergeometric clustering coefficients C_{vw}). Values refer to Fig. 1b in the main paper.

false neg. orthologs	r (P-value)	ρ (P-value)	s_{KS} (P-value)
-10%	$0.69\ (1.6 \times 10^{-2})$	$0.98\ (5.4 \times 10^{-3})$	$0.22\ (0.96)$
-30%	$0.65\ (2.8 \times 10^{-2})$	$0.93\ (8.3 \times 10^{-3})$	$0.17\ (0.99)$
-50%	$0.58\ (5.9 \times 10^{-2})$	$0.88\ (1.2 \times 10^{-2})$	$0.17\ (0.99)$
-70%	$0.41\ (0.44)$	$0.73\ (3.8 \times 10^{-2})$	$0.28\ (0.81)$
false pos. orthologs	r (P-value)	ρ (P-value)	s_{KS} (P-value)
$+10\%$	$0.69\ (1.7 \times 10^{-2})$	$0.95\ (7.2 \times 10^{-3})$	$0.17\ (0.99)$
$+30\%$	$0.73\ (9.3 \times 10^{-3})$	$0.98\ (5.4 \times 10^{-3})$	$0.22\ (0.96)$
$+50\%$	$0.75\ (5.6 \times 10^{-3})$	$0.98\ (5.4 \times 10^{-4})$	$0.22\ (0.96)$
$+70\%$	$0.79\ (2.7 \times 10^{-3})$	$0.93\ (2.4 \times 10^{-4})$	$0.22\ (0.96)$

Table 3. Pearson's (r), Spearman's rank correlation coefficients (ρ) and Kolmogorov-Smirnov scores (s_{KS}) of perturbed distributions of the excess retention ER_k of proteins that have an ortholog in *C. elegans*. Here, we mimicked the presence of false positive/negatives orthologs by randomly labeling up to 70%/removing up to 70% of proteins having orthologs in worm. Averaging over $1,000$ samples, correlation values presented suggest that the initial trend toward evolutionary conservation with elevated level of interaction prevails. Values refer to insets in Fig. 2a,b in the main paper.

false neg. links	r (P-value)	ρ (P-value)	s_{KS} (P-value)
−10%	0.87 (1.7×10^{-4})	0.97 (6.3×10^{-3})	0.11 (0.99)
−30%	0.86 (7.6×10^{-5})	0.97 (6.3×10^{-3})	0.11 (0.99)
−50%	0.87 (6.9×10^{-5})	0.97 (6.3×10^{-3})	0.11 (0.99)
−70%	0.87 (1.6×10^{-4})	0.92 (9.5×10^{-3})	0.11 (0.99)
**false pos. links**	**r (P-value)**	**ρ (P-value)**	**s_{KS} (P-value)**
+10%	0.87 (1.9×10^{-4})	0.97 (6.3×10^{-3})	0.22 (0.97)
+30%	0.87 (1.8×10^{-4})	(0.97×10^{-3})	0.27 (0.83)
+50%	0.87 (1.9×10^{-4})	0.97 (6.3×10^{-3})	0.39 (0.44)
+70%	0.87 (1.9×10^{-4})	0.97 (6.3×10^{-3})	0.39 (0.44)

Table 4. Pearson's (r), Spearman's rank correlation coefficients (ρ) and Kolmogorov-Smirnov scores (s_{KS}) of perturbed distributions of the excess retention ER_k of proteins that have an ortholog in *C. elegans*. Here, we mimicked the presence of false positive/negatives links by randomly adding up to 70% new interactions and deleting up to 70% previously present interactions. Here, we mimicked the presence of false positive/negatives links by randomly adding/removing up to 70% new/previoulsy present links. Averaging over $1,000$ samples, correlation values presented suggest that the initial trend toward evolutionary conservation with increased local clustering around links prevails. Values refer to Fig. 2a,b in the main paper.

false neg. orthologs	r (P-value)	ρ (P-value)	s_{KS} (P-value)
-10%	$0.79\ (7.6 \times 10^{-4})$	$0.94\ (3.1 \times 10^{-3})$	$0.09\ (1.0 \times 10^{-9})$
-30%	$0.83\ (1.5 \times 10^{-4})$	$0.96\ (2.3 \times 10^{-3})$	$0.14\ (1.3 \times 10^{-4})$
-50%	$0.85\ (6.8 \times 10^{-5})$	$0.96\ (2.3 \times 10^{-3})$	$0.14\ (1.3 \times 10^{-4})$
-70%	$0.87\ (2.1 \times 10^{-5})$	$0.96\ (2.3 \times 10^{-3})$	$0.18\ (0.11)$
**false pos. orthologs**	**r (P-value)**	**ρ (P-value)**	**s_{KS} (P-value)**
$+10\%$	$0.75\ (1.9 \times 10^{-3})$	$0.93\ (3.4 \times 10^{-4})$	$0.14\ (1.3 \times 10^{-4})$
$+30\%$	$0.81\ (3.3 \times 10^{-4})$	$0.95\ (2.5 \times 10^{-3})$	$0.14\ (1.3 \times 10^{-2})$
$+50\%$	$0.78\ (1.0 \times 10^{-3})$	$0.94\ (2.8 \times 10^{-3})$	$0.23\ (8.4 \times 10^{-2})$
$+70\%$	$0.85\ (6.1 \times 10^{-5})$	$0.96\ (2.3 \times 10^{-2})$	$0.32\ (0.42)$

Table 5. Pearson's (r), Spearman's rank correlation coefficients (ρ) and Kolmogorov-Smirnov scores (s_{KS}) of perturbed distributions of the excess retention $ER_{C_{vw}}$ of interacting pairs of proteins that both have an ortholog in *C. elegans* and the hypergeometric clustering coefficient of the link between them. Here, we mimicked the presence of false positive/negatives orthologs by randomly labeling up to 70%/removing up to 70% of proteins having orthologs in worm. Averaging over $1,000$ samples, correlation values presented suggest that the initial trend toward evolutionary conservation with increased local clustering around links prevails. Values refer to the insets of Fig. 2c,d in the main paper.

false neg. links	r (P-value)	ρ (P-value)	s_{KS} (P-value)
-10%	$0.92\ (4.4 \times 10^{-7})$	$0.96\ (2.3 \times 10^{-3})$	$0.14\ (1.1 \times 10^{-4})$
-30%	$0.92\ (7.5 \times 10^{-6})$	$0.98\ (5.4 \times 10^{-3})$	$0.20\ (2.4 \times 10^{-2})$
-50%	$0.91\ (5.4 \times 10^{-6})$	$0.92\ (6.0 \times 10^{-3})$	0.27 (0.26)
-70%	$0.91\ (2.1 \times 10^{-5})$	$0.78\ (2.7 \times 10^{-2})$	0.25 (0.14)
**false pos. links**	**r (P-value)**	**ρ (P-value)**	**s_{KS} (P-value)**
$+10\%$	$0.74\ (2.8 \times 10^{-3})$	$0.93\ (3.4 \times 10^{-3})$	$0.14\ (1.3 \times 10^{-4})$
$+30\%$	$0.71\ (5.2 \times 10^{-3})$	$0.93\ (3.4 \times 10^{-3})$	$0.14\ (1.4 \times 10^{-4})$
$+50\%$	$0.87\ (4.9 \times 10^{-5})$	$0.96\ (3.8 \times 10^{-3})$	$0.14\ (1.6 \times 10^{-4})$
$+70\%$	$0.95\ (1.0 \times 10^{-8})$	$0.98\ (1.9 \times 10^{-3})$	$0.14\ (1.0 \times 10^{-4})$

Table 6. Pearson's (r), Spearman's rank correlation coefficients (ρ) and Kolmogorov-Smirnov scores (s_{KS}) of perturbed distributions of the excess retention $ER_{C_{vw}}$ of interacting protein pairs that both have an ortholog in *C. elegans* and the hypergeometric clustering coefficient of the link between them. Here, we mimicked the presence of false positive/negatives links by randomly adding/removing up to 70% new/previously present links. Averaging over $1,000$ samples, correlation values presented suggest that the initial trend toward evolutionary conservation with increased local clustering around links prevails. Values refer to Fig. 2c,d in the main paper.

threshold	*S. cerevisiae* t (P-value)	*P. falciparum* t (P-value)
0	31.3 (1.7×10^{-214})	36.1 (9.1×10^{-285})
1	35.8 (8.9×10^{-281})	38.9 $(< 10^{-350})$
2	36.5 (2.5×10^{-291})	38.2 (1.5×10^{-319})
3	36.1 (3.7×10^{-285})	37.6 (3.0×10^{-309})
4	37.0 (7.4×10^{-299})	36.3 (1.1×10^{-288})

Table 7. Students t-test of frequency distributions of coexpression correlations in *S. cerevisiae* and *P. falciparum.* The individual distributions were compared with organism specific background distributions (initial, Fig. 3a,b) that embrace all protein pairs. The values refer to Fig. 3a,b

.

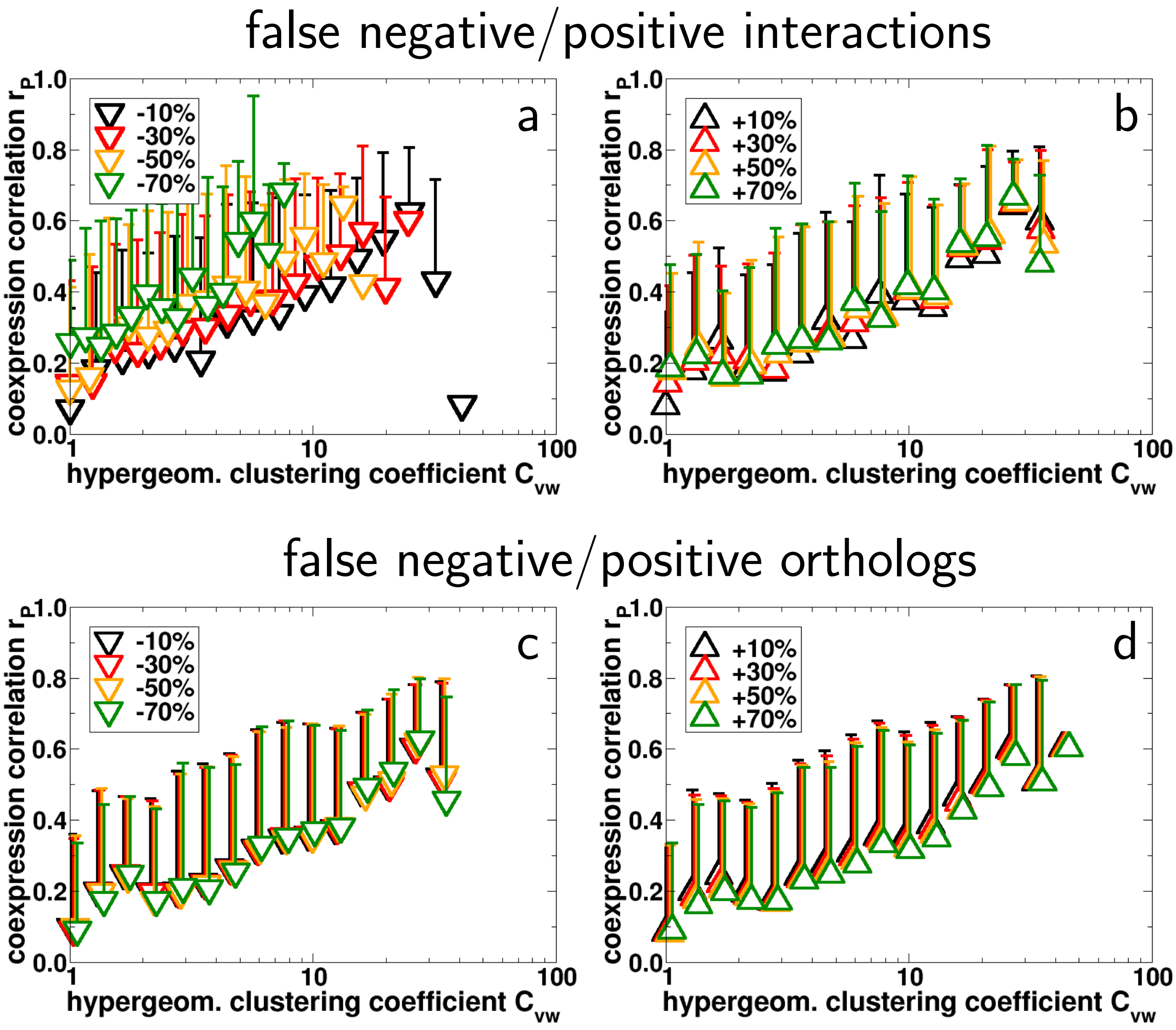

Figure 1. Perturbation analysis of coexpression patterns in Yeast. **(a)** In order to assess the impact of severely inconsistent Yeast protein interaction data on the correlation between hypergeometric clustering coefficient C_w and coexpression correlation r_P, we removed 10-70% of interactions between randomly selected protein pairs, mimicking false negatives. **(b)** Simulating the effects of false positives, we randomly added 10-70% more interactions than originally present in the network. In each case, we logarithmically binned data points of $1,000$ repeats according to their hypergeometric clustering coefficient and determined the average coexpression coefficient among interacting Yeast proteins that have an ortholog in *P. falciparum*. In both cases, we observe that the initial ascending trend we already encountered in Fig. 3c in the main paper prevails. Significant Kolmogorv-Smirnov scores determined by comparing perturbed and the initial distributions (Table 7) support our observations. **(c)** We repeated these perturbation analysis for the consequences of false negative orthologs by eliminating 10-70% of the proteins present in the set of Plasmodium orthologs. **(d)** Mimicking the presence of false positive orthologs we labeled 10-70% more proteins as orthologs in Plasmodium that were originally present. In all cases, we observe that the initial (empirically derived) ascending trend prevails, results which are further supported by strong and significant Pearson's and Spearman's rank correlation coefficients as well as Kolmogorov-Smirnov scores (Table 7).

false neg./pos. interactions	r (P-value)	ρ (P-value)	s_{KS} (P-value)
−10%	0.82 (9.7×10^{-6})	0.66 (1.1×10^{-2})	0.13 (0.99)
−30%	0.87 (2.3×10^{-7})	0.97 (1.8×10^{-4})	0.21 (0.88)
−50%	0.78 (4.1×10^{-5})	0.92 (3.6×10^{-4})	0.29 (0.52)
−70%	0.93 (2.8×10^{-10})	0.92 (3.6×10^{-4})	0.50 (0.04)
+10%	0.92 (1.1×10^{-9})	0.94 (4.2×10^{-4})	0.19 (0.99)
+30%	0.93 (1.5×10^{-10})	0.97 (2.9×10^{-4})	0.16 (0.99)
+50%	0.91 (1.3×10^{-8})	0.95 (3.6×10^{-4})	0.16 (0.99)
+70%	0.86 (1.0×10^{-6})	0.94 (4.4×10^{-4})	0.13 (0.99)
**false neg./pos. orthologs**	**r (P-value)**	**ρ (P-value)**	**s_{KS} (P-value)**
−10%	0.92 (4.6×10^{-9})	0.97 (2.9×10^{-4})	0.49 (0.03)
−30%	0.91 (1.1×10^{-8})	0.96 (3.4×10^{-4})	0.49 (0.04)
−50%	0.92 (2.4×10^{-9})	0.96 (3.4×10^{-4})	0.48 (0.05)
−70%	0.86 (1.1×10^{-6})	0.95 (3.9×10^{-4})	0.48 (0.05)
+10%	0.91 (6.3×10^{-9})	0.96 (2.0×10^{-4})	0.5 (0.03)
+30%	0.92 (1.5×10^{-9})	0.97 (1.6×10^{-4})	0.5 (0.03)
+50%	0.93 (2.5×10^{-10})	0.97 (1.6×10^{-4})	0.5 (0.03)
+70%	0.93 (9.7×10^{-11})	0.98 (1.4×10^{-4})	0.5 (0.03)

Table 8. Pearson's (r), Spearman's rank correlation coefficients (ρ) and Kolmogorov-Smirnov scores (s_{KS}) of perturbed dependencies between hypergeometric clustering coefficient and coexpression coefficient of Yeast as presented in Fig. 1 of the Supplementary Material.

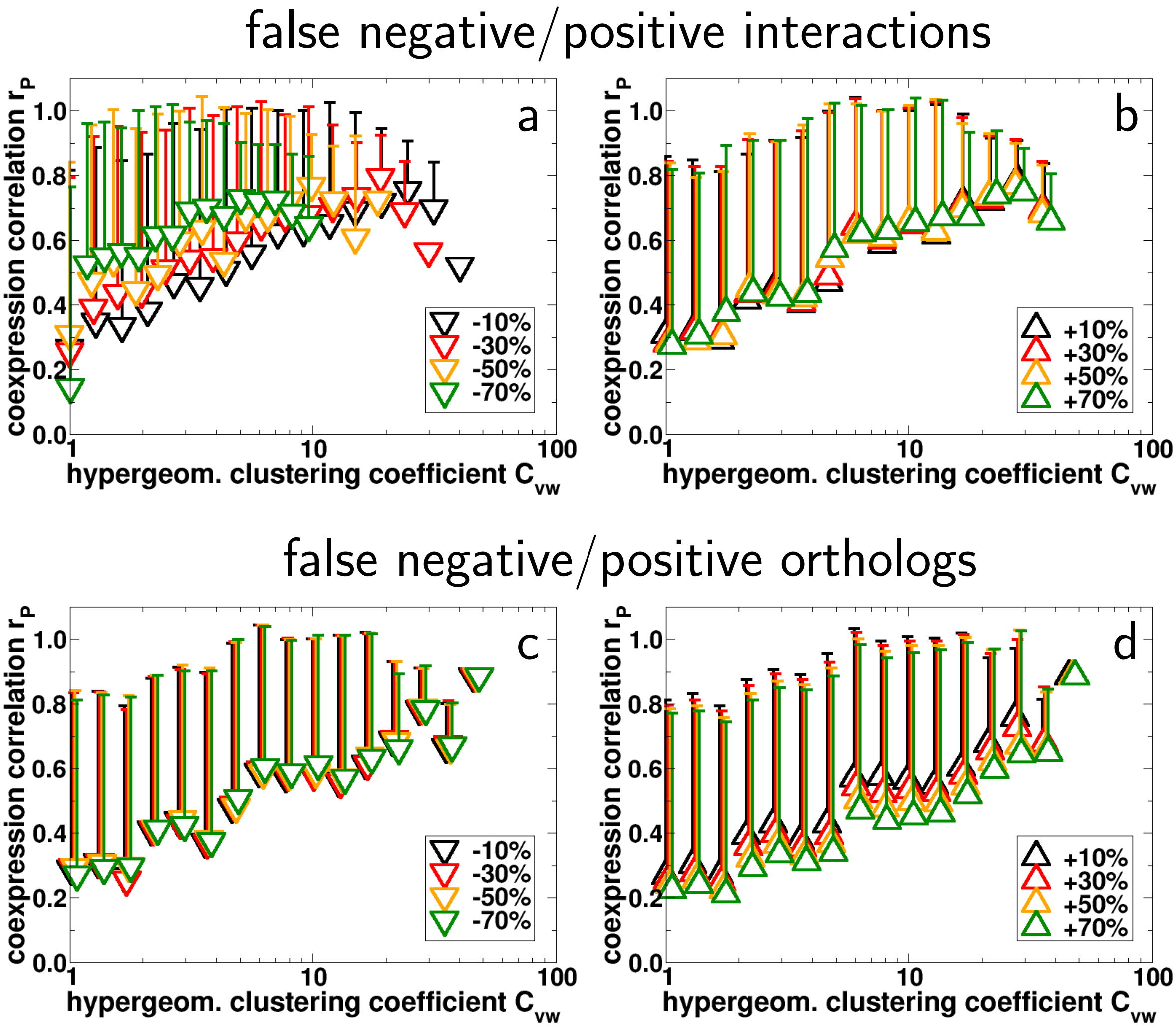

Figure 2. Perturbation analysis of coexpression patterns in Plasmodium. **(a)** In order to assess the impact of severely inconsistent protein interaction data on the correlation between hypergeometric clustering coefficient C_w and coexpression correlation r_P in Plasmodium, we removed 10-70% of interactions between randomly selected Yeast protein pairs, mimicking false negatives. **(b)** Simulating the effects of false positives, we randomly added 10-70% more interactions than originally present in the Yeast network. In each case, we logarithmically binned data points of $1,000$ repeats according to their hypergeometric clustering coefficient. Assuming that the interactions among orthologous Yeast proteins are conserved in Plasmodium we averaged the Plasmodium specific coexpression coefficients in each bin. In both cases, we observe that the initial ascending trend we already encountered in Fig. 3c in the main paper prevails. Significant Kolmogorov-Smirnov scores determined by comparing perturbed and the initial distributions (Table 8) support our observations. **(c)** We repeated these perturbation analysis for the consequences of false negative orthologs by eliminating 10-70% of the proteins present in the set of Plasmodium orthologs. **(d)** Mimicking the presence of false positive orthologs we labeled 10-70% more proteins as orthologs in Plasmodium that were originally present ((b) inset). In all cases, we observe that the initial (empirically derived) ascending trend prevails, results which are further supported by strong and significant Pearson's and Spearman's rank correlation coefficients as well as Kolmogorov-Smirnov scores (Table 8).

false neg./pos. links	r (P-value)	ρ (P-value)	s_{KS} (P-value)
−10%	0.61 (6.1×10^{-3})	0.88 (6.5×10^{-4})	0.49 (4.8×10^{-2})
−30%	0.61 (7.1×10^{-3})	0.87 (6.8×10^{-4})	0.54 (2.0×10^{-2})
−50%	0.69 (1.5×10^{-3})	0.87 (1.2×10^{-3})	0.70 (1.0×10^{-3})
−70%	0.56 (1.7×10^{-2})	0.81 (2.3×10^{-3})	0.83 (4.3×10^{-2})
+10%	0.81 (2.8×10^{-5})	0.94 (4.4×10^{-4})	0.47 (7.1×10^{-2})
+30%	0.80 (3.2×10^{-5})	0.94 (4.0×10^{-4})	0.43 (0.11)
+50%	0.79 (6.8×10^{-5})	0.96 (3.4×10^{-4})	0.47 (7.0×10^{-2})
+70%	0.75 (2.7×10^{-4})	0.95 (3.6×10^{-4})	0.50 (4.2×10^{-2})
false neg./pos. orthologs	r (P-value)	ρ (P-value)	s_{KS} (P-value)
−10%	0.88 (1.1×10^{-7})	0.94 (2.4×10^{-4})	0.43 (0.12)
−30%	0.88 (1.1×10^{-7})	0.94 (2.6×10^{-4})	0.45 (0.08)
−50%	0.88 (9.5×10^{-8})	0.96 (2.0×10^{-4})	0.48 (0.05)
−70%	0.87 (3.5×10^{-7})	0.97 (1.8×10^{-4})	0.45 (0.08)
+10%	0.89 (2.3×10^{-8})	0.95 (2.2×10^{-4})	0.42 (0.13)
+30%	0.88 (2.1×10^{-7})	0.94 (4.2×10^{-4})	0.37 (0.26)
+50%	0.94 (2.6×10^{-11})	0.96 (1.9×10^{-4})	0.34 (0.33)
+70%	0.96 (2.8×10^{-13})	0.97 (1.7×10^{-4})	0.28 (0.59)

Table 9. Pearson's (r), Spearman's rank correlation coefficients (ρ) and Kolmogorov-Smirnov scores (s_{KS}) of perturbed dependencies between hypergeometric clustering coefficient and coexpression coefficient in Plasmodium as presented in Fig. 2 of the Supplementary Material.
